# Diagnostic Accuracy of FebriDx: A Rapid Test to Detect Immune Responses to Viral and Bacterial Upper Respiratory Infections

**DOI:** 10.3390/jcm6100094

**Published:** 2017-10-07

**Authors:** Wesley H. Self, Jeffrey Rosen, Stephan C. Sharp, Michael R. Filbin, Peter C. Hou, Amisha D. Parekh, Michael C. Kurz, Nathan I. Shapiro

**Affiliations:** 1 Department of Emergency Medicine, Vanderbilt University Medical Center, Nashville, TN 37232, USA; wesley.self@vanderbilt.edu; 2 Clinical Research of South Florida, Coral Gables, FL 33134, USA; jrosen@crsouthflorida.com; 3 Clinical Research Associates, Inc., Nashville, TN 37203, USA; ssharp@clinicalresearchassociates.com; 4 Department of Emergency Medicine, Massachusetts General Hospital, Boston, MA 02114, USA; mfilbin@partners.org; 5 Department of Emergency Medicine, Brigham and Women’s Hospital, Boston, MA 02115, USA; phou@partners.org; 6 Department of Emergency Medicine, New York Methodist Hospital, Brooklyn, NY 11215, USA; parekh@doctoris.org; 7 Department of Emergency Medicine, University of Alabama School of Medicine, Birmingham, AL 35233, USA; mckurz@uabmc.edu; 8 Department of Emergency Medicine, Beth Israel Deaconess Medical Center, Boston, MA 02215, USA

**Keywords:** respiratory infection, antibiotic stewardship, MxA, CRP, immunoassay, FebriDx

## Abstract

C-reactive protein (CRP) and myxovirus resistance protein A (MxA) are associated with bacterial and viral infections, respectively. We conducted a prospective, multicenter, cross-sectional study of adults and children with febrile upper respiratory tract infections (URIs) to evaluate the diagnostic accuracy of a rapid CRP/MxA immunoassay to identify clinically significant bacterial infection with host response and acute pathogenic viral infection. The reference standard for classifying URI etiology was an algorithm that included throat bacterial culture, upper respiratory PCR for viral and atypical pathogens, procalcitonin, white blood cell count, and bandemia. The algorithm also allowed for physician override. Among 205 patients, 25 (12.2%) were classified as bacterial, 53 (25.9%) as viral, and 127 (62.0%) negative by the reference standard. For bacterial detection, agreement between FebriDx and the reference standard was 91.7%, with FebriDx having a sensitivity of 80% (95% CI: 59–93%), specificity of 93% (89–97%), positive predictive value (PPV) of 63% (45–79%), and a negative predictive value (NPV) of 97% (94–99%). For viral detection, agreement was 84%, with a sensitivity of 87% (75–95%), specificity of 83% (76–89%), PPV of 64% (63–75%), and NPV of 95% (90–98%). FebriDx may help to identify clinically significant immune responses associated with bacterial and viral URIs that are more likely to require clinical management or therapeutic intervention, and has potential to assist with antibiotic stewardship.

## 1. Introduction

Acute respiratory infections (ARI) are the most common reason for oral antibiotic prescriptions in the United States [1]. Recent data suggest that approximately 30% of antibiotics used in the outpatient setting are inappropriate, largely driven by misuse of antibiotics for viral upper respiratory tract (URI) infections [2]. Overuse of antibiotics has been linked to several negative outcomes, including development of antibiotic resistance, antibiotic-associated infections, increased costs, and drug toxicities [3]. Antibiotic stewardship programs focus on guiding clinicians to use antibiotics for URIs only when adequate evidence for bacterial infection exists [4]. However, rapid diagnostic tests to assist clinicians in the identification of bacterial respiratory infections are lacking, which has hampered antibiotic stewardship efforts [5].

FebriDx^®^ (RPS Diagnostics; Sarasota, FL, USA) is a new point-of-care diagnostic test designed to rapidly identify host immune responses associated bacterial and viral infections, with the goal of providing clinicians real-time, accurate information to assist with antibiotic prescribing decisions for URIs [6,7]. FebriDx provides qualitative results for elevated serum levels of c-reactive protein (CRP), an acute phase reactant associated with bacterial infection, and myxovirus resistance protein A (MxA), which is a derivative of interferonα/β associated with viral infection [8,9]. The purpose of this study was to evaluate the diagnostic accuracy of the FebriDx test for identifying immune responses associated with bacterial and viral URIs in patients with acute fever and URI symptoms that are more likely to require clinical management or therapeutic intervention from those who present with a less clinically significant microbiologically unconfirmed respiratory illness (MURI).

## 2. Experimental Section

### 2.1. Design and URI Study Population

This was a prospective, cross sectional, observational study using a convenience sample of febrile patients with clinical signs and symptoms of a URI. Children and adults were enrolled between January and November 2014 at 10 clinical sites in the US, including 7 academic emergency departments, 2 community urgent care centers, and 1 ambulatory clinical research site. Major inclusion criteria included: age >1 year, new fever ≥ 100.5 °F within the past 3 days, and new onset of cough or sore throat within the past 7 days. Major exclusion criteria included recent history of trauma or surgery, use of antibiotics, antiviral agents, interferon therapy, immunosuppressive therapy or a live viral immunization within the past 30 days. Full eligibility is listed in Supplemental Table S1. The study was approved by the governing institutional review board for each enrolling center. Written informed consent was obtained from each participant or their legal authorized representative, as appropriate.

### 2.2. FebriDx Measurements

Each patient underwent testing with FebriDx, which is a rapid, qualitative, single-use, disposable, whole blood immunoassay with a turn-around time of 15 min [6]. FebriDx requires 5 mcL of whole blood obtained by a capillary (“finger”) stick. It provides qualitative results for elevated levels of CRP (≥20 mg/L) and MxA (≥40 ng/mL). There is no current universal MxA ELISA standard and the MxA ELISA was used to determine the medical decision point for the FebriDx MxA result line. The standard material in the BioVender MxA ELISA (BioVender; Brno, Czech Republic) uses a truncated recombinant MxA construct, which is less prone to aggregation. The assay conditions also contain a high salt content to ensure monomeric MxA. Taken together, these factors tend to dramatically enhance the accuracy and more importantly the reproducibility of an assay and explain why the FebriDx MxA medical decision points are near 40 ng/mL versus 235 ng/mL as seen on other MxA ELISA tests [10]. FebriDx results indicating an elevated CRP without an associated elevated MxA were interpreted as bacterial infection. FebriDx results indicating elevated MxA, regardless of CRP level, were interpreted as viral infection. Valid tests showing no elevated CRP or MxA were interpreted as negative.

Study personnel at the site obtained a whole blood sample by finger stick, performed FebriDx testing according to the manufacturer’s instructions [6], and interpreted the results as bacterial, viral or negative. Personnel performing FebriDx testing were blinded to all reference testing outlined below. FebriDx results were not used for clinical care.

### 2.3. Outcome

**Figure 1:**
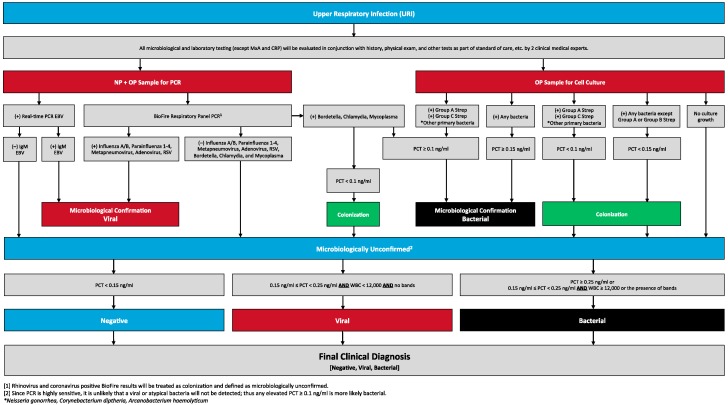
Reference testing algorithm for guidance on classifying bacterial and viral infections with a systemic immune response.

We used a reference-testing algorithm combined with a physician panel over-read as the reference standard for identifying the type of infection present (Figure 1). There were 2 outcomes of interest: acute bacterial infection with host response and an acute pathogenic viral infection. Procalcitonin (PCT) and white blood cell (WBC) count were used in the reference standard to identify a host response [10,11]. Viruses that are typically pathogenic when detected in the upper respiratory tract (listed below) were classified as an acute viral infection [12,13,14]. Meanwhile, viruses that commonly colonize the respiratory tract without causing symptoms, such as rhinovirus, or produce latent infections with periodic DNA shedding, such as Herpes Simplex Virus (HSV), were not considered acute pathogenic infections in this study [12,13,14].

### 2.4. Reference Testing Algorithm

The reference testing algorithm is illustrated in Figure 1. Each patient underwent the following 6 diagnostic tests: (1) throat swab bacterial culture; (2) multiplex polymerase chain reaction (PCR) of a combined nasopharyngeal and oropharyngeal (NP/OP) sample using the FilmArray^®^ Respiratory Panel (BioMerieux, Inc.; Marcy-l’Etoile, France) [15]; (3) real-time reverse transcriptase PCR of an NP/OP sample for Epstein-Barr Virus (EBV); (4) EBV IgM serum antibody with the Immunosimplicity^®^ IS-EBV-VCA IgM Test Kit (Diamedix Co; Miami Lakes, FL, USA) [16]; (5) serum PCT concentration measurement with the BRAHMS PCT KRYPTOR Analyzer (Thermo Fisher; Waltham, MA, USA) [17]; (6) WBC count with band differential percentage, and (7) MxA protein ELISA and high sensitivity c-reactive protein enzyme immunoassay (Biocheck; Foster City, CA, USA). Reference testing was completed at a central laboratory and blinded to patients, treating clinicians, and study personnel who performed FebriDx testing.

The reference testing algorithm classified patients as having a bacterial infection if any of the following 5 criteria were met: (1) throat culture positive for a bacteria that commonly causes pharyngitis (Group A and C β-hemolytic *Streptococci*, *N. gonorrhoeae*, *C. diphtheria*, *A. haemolyticum*) plus PCT ≥0.1 ng/mL; (2) throat culture positive for any other bacteria plus PCT ≥0.15 ng/mL; (3) NP/OP sample PCR positive for atypical bacteria (*M. pneumoniae*, *C. pneumoniae*, *B. pertussis*) plus PCT ≥0.1 ng/mL; (4) PCT ≥0.25 ng/mL plus no identified pathogen; (5) PCT ≥0.15 ng/mL plus WBC ≥15,000 cells/mcL or presence of WBC bands plus no identified pathogen [18,19,20,21,22]. Meanwhile, the reference testing algorithm classified patients as having a viral infection if any of the following 3 criteria were met: (1) NP/OP sample PCR positive for influenza A or B, adenovirus, respiratory syncytial virus (RSV), human metapneumovirus, or parainfluenza viruses 1–4; (2) NP/OP sample PCR positive for EBV plus serum IgM positive for EBV; (3) PCT between 0.15 ng/mL and 0.25 ng/mL plus WBC <15,000 cells/mcL plus no WBC bands plus no identified pathogen [21]. If a patient met criteria for both a viral and bacterial infection, the patient was classified as bacterial. Patients who did not meet any of these criteria for bacterial or viral infection were classified as negative by the reference testing algorithm.

This study examined outpatient URI and the reference method was developed to help account for microbial colonization. The reference value of PCT in adults and children older than 72 h is 0.15 ng/mL or less [23]. The majority of PCT data in the literature is related to bacteremic patients, sepsis and those with lower respiratory tract infections. Stolz et al., examined PCT cutoffs for a variety of respiratory infections and demonstrated that a PCT of 0.1 ng/mL has 94% sensitivity for bacterial infection and 72% specificity while at 0.25 ng/mL cutoff, the sensitivity for bacterial infection decreased to 84% while the specificity increased to 98% [24]. When specifically looking at URI conditions, such as pharyngitis, both Elsammak and Christiansen et al., showed that PCT levels ranged between 0.1 ng/mL to 6 ng/mL with the median values found between 0.1 ng/mL and 0.3 ng/mL [25,26]. In the context of a negative FilmArray^®^ Respiratory Panel test for the most common viral and atypical bacterial pathogens and an associated elevated WBC or the presence of bands, an elevated PCT level of 0.1 ng/mL is more likely to be a bacterial infection than when it is measured across the entire patient population.

### 2.5. Physician Panel Over-Read

Two physicians with expertise in respiratory infections reviewed each case in detail. These reviews used the reference testing algorithm classification (bacterial, viral, or negative) as a guideline, but also included a review of all clinical and laboratory information available from the study case report form including the results from throat culture, molecular respiratory pathogen panels, and the following blood tests: CBC including WBC and a diff with bands and lymphocytes, procalcitonin, Epstein-Barr virus IgM/IgM as well as additional standard of care tests performed including rapid flu and rapid strep tests. The 2 physicians reviewed each case together and came to a consensus classification of bacterial, viral, or negative. This physician over-read could lead to reclassification compared to the initial classification from the algorithm. Thus, the final reference standard classification (bacterial, viral, or negative) consisted of an algorithm-guided consensus decision by 2 physicians who were blinded to the FebriDx results.

### 2.6. Statistical Analysis

Initially, FebriDx and reference standard results were analyzed using a three-category classification scheme (bacterial vs viral vs negative). Agreement between FebriDx and the reference standard was calculated using the unweighted Cohen’s Kappa statistic [27]. Next, we separately evaluated FebriDx accuracy for bacterial and viral detections. For the bacterial analysis, FebriDx and reference standard results were classified into bacterial vs not bacterial dichotomous categories; for this analysis, viral and negative results were collapsed into a “not bacterial” group. For the viral analysis, results were reclassified as viral vs not viral, with the bacterial and negative results collapsed into a “not viral” group. Sensitivity, specificity, and positive and negative predictive values were separately calculated for bacterial and viral detection. These calculations were also repeated after stratifying of the study population into 3 age groups (<18 years; 18–50 years; and >50 years).

Confidence intervals for sensitivity, specificity, and predictive values were calculated using the binomial exact method. Confidence intervals for kappa statistics were calculated by bootstrap with 1000 replications [28]. Statistical analyses were conducted with Stata 12 (StataCorp, College Station, TX, USA).

### 2.7. Asymptomatic Control Population

In addition to the URI population, we also enrolled a convenience sample of asymptomatic control participants to test FebriDx specificity in people without any signs or symptoms of infection. Major eligibility criteria for the asymptomatic control population included: age >1 year, and no fever, cough, chills, rhinorrhea, or sore throat within the past 14 days. Full eligibility criteria are displayed in Supplemental Table S2. FebriDx measurements were completed on asymptomatic controls as described above for the URI population. Asymptomatic control participants did not undergo reference testing and were assumed to have a “negative” etiology for the reference standard. These patients were tested with an MxA protein ELISA and high sensitivity c-reactive protein enzyme immunoassay. This asymptomatic control population was analyzed separately from the primary URI study population. Specificity (true negative rate) and the false positive rate for FebriDx were calculated.

## 3. Results

### 3.1. URI Study Population

During the study period, we enrolled 206 febrile URI patients. All enrolled URI patients underwent FebriDx testing and had a valid FebriDx result. One (0.5%) patient did not have adequate testing for the reference standard and was excluded, resulting in a final population of 205 URI patients for analysis. This population included 56 (27.3%) children <18 years old and 149 (72.7%) adults (Table 1).

#### 3.1.1. Reference Standard Results

Application of the reference standard, including the reference testing algorithm followed by physician panel over-read, resulted in classifying 25 (12.2%) patients as bacterial, 53 (25.9%) as viral and 127 (62.0%) as negative (Table 2). The physician panel agreed with classification assigned by the algorithm for 203 (99.0%) patients. Two patients were reclassified by the physician panel as more likely bacterial infections. The first patient had a throat culture positive for heavy growth of Group A beta hemolytic *Streptococcus* (GABHS) and a PCT value of 0.09 ng/mL that was thought to more likely represent a bacterial infection in the presence of GABHS culture and a negative viral PCR panel, this was deemed more likely to be a bacterial infection. The second case demonstrated a negative microbiology testing but an elevated PCT of 0.14 ng/mL and elevated WBC count of 10.5 thousand cells/mcl (Table 2).

Among the 25 bacterial cases, 13 (52.0%) were microbiologically confirmed, while 12 were classified as bacterial based on biomarkers measurements (Table 2). Among the 53 viral cases, 50 (94.3%) were microbiologically confirmed. Of these 50 patients with a microbiologically-confirmed viral infection, 42 (83%) had PCT <0.1 ng/mL, 5 (10%) had PCT between 0.1 ng/mL and 0.25 ng/mL, and 3 (6%) had PCT ≥0.25 ng/mL. Overall, the most common pathogens were influenza (*n* = 33), parainfluenza (*n* = 9), and GABHS (*n* = 7).

#### 3.1.2. Diagnostic Accuracy of FebriDx

**Figure 2:**
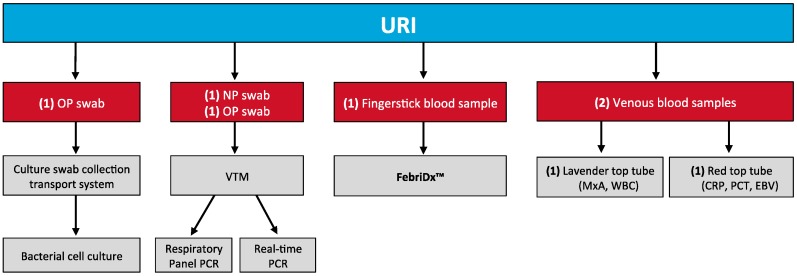
Flow diagram of patient participation in the upper respiratory tract infection population.

FebriDx classified 32 (15.6%) patients as bacterial, 72 (35.1%) as viral, and 101 (49.3%) as negative (Table 3; Figure 2). Using this three-category classification scheme, overall agreement between FebriDx and the reference standard algorithm was 76.6% (Kappa: 0.60; 95% CI: 0.50, 0.69). When classifying results as bacterial vs not bacterial, overall agreement was 91.7%, with FebriDx sensitivity of 80% (95% CI: 59–93%), specificity of 93% (89–97%), positive predictive value (PPV) of 63% (45–79%), and a negative predictive value (NPV) of 97% (94–99%). Diagnostic accuracy for bacteria was similar across age groups (Table 4A). Among the 7 patients with positive throat cultures for GABHS, 6 (85.7%) had a bacterial result by FebriDx.

Five patients had false negative FebriDx results for bacterial detection. One of these patients had a throat culture positive for GABHS with PCT of 0.1 ng/mL. The second patient had an NP/OP PCR positive for *Chlamydophila pneumoniae* with PCT of 0.73 ng/mL. The remaining 3 patients had negative bacterial cultures and PCR tests, but were classified as bacterial by the reference algorithm due to PCT concentrations between 0.15 ng/mL and 0.25 ng/mL with a concurrent white blood cell count > 15 thousand cells/mcL (1 patient) or the presence of bands (2 patients).

When classifying results as viral vs not viral, FebriDx and the reference standard had 84% overall agreement, with FebriDx sensitivity of 87% (95% CI: 75–95 specificity of 83% (76–89%), PPV of 64% (63–75%), and NPV of 95% (90–98%). There was a trend noted toward higher sensitivity and lower specificity in children compared with adults (Table 4B). Of note, among 33 patients with influenza detected by RT-PCR, 29 (88%) had a viral result by FebriDx. If rhinovirus and coronavirus are included in the analysis and considered positive only when the MxA ELISA is positive to help differentiate against asymptomatic viral carriage, the FebriDx viral performance values showed a sensitivity of 86% (75–94), specificity of 88% (76–88), PPV of 78% (52–74), and NPV of 93% (90–97).

A small subset of 26 patients had a documented versus a reported fever. The rate of microbiologically unconfirmed cases was 28% and the test demonstrated 100% (4/4) bacterial sensitivity, 95% (21/22) specificity, 82% (9/11) viral sensitivity, and 87% (13/15) viral specificity in this population.

#### 3.1.3. Asymptomatic Control Results

Study personnel enrolled 165 people as asymptomatic control subjects. Two (1.2%) of these subjects were excluded due to invalid FebriDx results, leaving 163 subjects for analysis. Effort was made to enroll a representative population however only a limited number of pediatric patients <18 years were included in this arm of the study. Demographics of the asymptomatic control subjects were similar to those in the URI population (Table 1).

FebriDx results were negative for 161 of the 163 asymptomatic control subjects (specificity: 99%; 95% CI: 96–100%). FebriDx resulted in 2 false positive tests (false positive rate: 1.2%; 95% CI: 0.1–4.4%), with 1 false positive viral result and 1 false positive bacterial result. The false positive FebriDx viral result was confirmed with the MxA ELISA test < 40 ng/ml. The FebriDx false positive bacterial result was also shown to be an accurate measurement with an elevated CRP ≥ 20 mg/L confirmed by the CRP ELISA testing. One additional patient had an elevated CRP but was FebriDx negative. All other MxA ELISA and CRP ELISA tests were within the normal range.

## 4. Discussion

This manuscript describes a multi-center evaluation of FebriDx, a new point-of-care diagnostic test designed to rapidly provide clinicians with actionable, bedside data on the likely etiology of URIs. Using a small sample of capillary whole blood, FebriDx provides measurements of CRP and MxA, biomarkers that are associated with bacterial and viral infections, respectively [8,9]. In this prospective, multicenter study of 205 outpatients with febrile URIs and 163 asymptomatic controls, FebriDx was sensitive and specific for detection of bacteria with a host immune response and pathogenic viruses, with overall sensitivities and specificities in the 80–93% range. A limited number of patients less than 18 years old were enrolled with a bacterial infection making it challenging to compare sensitivity and specificity values to the other age groups. However, viral infections were more equally distributed among the age groups. Comparatively, the sensitivity of the FebriDx test was found to be higher in children with a lower specificity compared to the other age groups. This suggests that children have some unknown factor that leads to elevated MxA or a cross-reactivity with the anti-MxA antibody, or more likely, is partially related to the active exclusion of rhinovirus and coronavirus as shown above and based on the limitation of the FilmArray^®^ Respiratory Panel for detecting other viral pathogens such as parechoviruses, human bocavirus, and specifically, enterovirus-68 which produced a U.S. epidemic during the dates of the trial [16]. While the clinical utility of this promising technology will be further defined with additional study, FebriDx may serve as an important tool for future efforts at antibiotic stewardship, which have historically been hampered by a lack of rapid and accurate diagnostic tests to help clinicians distinguish between viral and bacterial infection [1,2,4,5].

Particular strengths of the study included: (1) the prospective, multicenter design; (2) standardized procedures and definitions used across all sites; (3) appropriate blinding of FebriDx and reference standard results to personnel who made FebriDx and reference testing measurements; and (4) inclusion of an asymptomatic control population to confirm high specificity of FebriDx in an asymptomatic population. Furthermore, in the current study, FebriDx correctly identified 86% of patients with GABHS pharyngitis as bacterial and 90% if inclusive of GCBHS, as well as 88% of patients with acute influenza as viral. This accuracy compares favorably with rapid antigen detection tests for GABHS [29,30] and influenza [31,32,33] which clinicians routinely use to assist with decisions about prescribing antibiotics and antiviral agents.

FebriDx provides qualitative measurements (high vs not high) of CRP and MxA. CRP is an acute phase protein that has been linked to the presence and severity of bacterial infection in numerous studies during the past 2 decades [9,34,35]. CRP concentrations >20 mg/L trigger a positive CRP reading on the FebriDx device. Previous studies suggest that patients with infectious respiratory symptoms and a CRP below this 20 mg/L threshold are likely to have non-bacterial, or self-limited infections [9,34,35,36,37,38]. CRP levels above this threshold identify a clinically significant immune response but cannot reliably differentiate between viral and bacterial etiology [8]; therefore, FebriDx also includes MxA as a marker of viral infection. MxA is a derivative of interferon-α and -β (type I interferons), and, like interferons, is upregulated in the presence of viral infection [8,39]. MxA has broad antiviral activity against RNA and DNA viruses [40]. The long serum half-life of MxA, with elevated levels detectable up to 10 days after viral infection, provides a distinct advantage for use as a biomarker compared to interferons, which have more variable and transient elevations in response to viral infection [39,41]. MxA concentrations ≥40 ng/mL trigger a positive reading on FebriDx, a level that has been identified as a sensitive threshold for identifying viral infections [8,36,42]. By combining CRP and MxA into a single test, FebriDx includes sensitive markers of both bacterial and viral infection.

In the current study, we sought to evaluate the accuracy of FebriDx for identifying clinically-significant acute bacterial and viral URIs. Therefore, we developed a reference standard algorithm that incorporated multiple individual tests with the aim of identifying bacterial infections with a host response and pathogenic viral infections (Figure 1). Recognizing that all clinically-important bacterial URIs were unlikely to be detected by throat culture and NP/OP PCR, we classified culture- and PCR-negative patients as bacterial when elevated PCT and WBC levels suggested bacterial infection [18,19,20,21,22]. Given the strong association between NP/OP PCR detection of influenza, adenovirus, parainfluenza, RSV, human metapneumovirus and symptomatic disease, [12,13,14] identification of any of these viruses was considered a pathogenic infection in our algorithm. Detection of other viruses that are more likely to represent asymptomatic detection (colonization/carriage), such as rhinovirus or coronavirus, were not considered pathogenic in the algorithm [12,13,14].

Our final reference standard for etiology (bacterial vs viral vs negative) also included a physician over-read of algorithm results to allow for clinically-logical classification. The physician over-read led to re-classification in only 1% of cases.

Similar to other work on respiratory infections [11,43,44] our study lacked a definitive reference standard for classifying viral and bacterial etiology. In fact, lack of definitive etiologic testing for respiratory infections has been recognized as a major unmet need in medicine and a central challenge for evaluating new diagnostic tests in this field [5]. We acknowledge our algorithm-based reference standard for identifying clinically-significant viral and bacterial URIs was imperfect and additional testing could be incorporated. For example, the algorithm did not include testing for bocavirus or human immunodeficiency virus (HIV). However, we believe this transparent, standardized, literature-based approach is useful and provides structured, reproducible results in a field devoid of superior alternatives.

While it is well known that viral infections may alter the oropharyngeal and nasopharyngeal host equilibrium and lead to bacterial growth and enhanced colonization, this does not necessarily lead to clinically significant infection [45]. Pathogen specific isolation with antigen, culture, or molecular testing cannot discriminate between colonization or the carrier state [46,47]. Differentiation of infection from colonization or a clinically insignificant local infection requires the demonstration of a systemic antibody response although this immune response is clinically impractical because it is both time-delaying and may lead to false-negative results following appropriate antibiotic therapy [48]. Other studies have shown that only 40–50% of the children with GABHS isolated from the upper respiratory tract, who presented with symptoms of tonsillitis or pharyngitis, demonstrated an immune response [49,50,51]. Ivaska et al., showed that in 83 patients presenting with pharyngitis, GABHS alone was found in 8 (9.6%), GABHS together with virus in 11 (13.3%), group C or G b-hemolytic streptococci (GCBHS, GGBHS) alone in two (2.4%), GCBHS or GGBHS together with virus in three (3.6%), and one or more viruses alone in 49 (59.0%) cases. Only 5 patients showed a 2-fold anti-streptolysin-O antibody increase in paired serum samples and of the 5 patients with an antibody response, three of them were GABHS positive, one of them was GCBHS positive, and one was negative for streptococci by throat culture. Blood MxA levels were found to be elevated in 80% of patients with viral pharyngitis, and remained low in 90% of patients with GABHS without virus detection [52].

In the current study, if both a viral and bacterial pathogen were microbiologically confirmed as described above in a single subject, the subject was characterized as a true bacterial infection. While a co-infection cannot be ruled out when both MxA and CRP are elevated, this condition was not seen in the current clinical trial. We defined a true co-infection as requiring the presence of a confirmed virus by PCR and the presence of bacterial cell culture growth or PCR positivity for an atypical bacterial pathogen, in association with a PCT elevation greater than ≥0.25 ng/mL and/or elevated WBC ≥ 12,000. Using this definition, no co-infections were confirmed. This low association of bacterial co-infection in patients with URI is consistent with the low 2% co-infection rate found by Mäkelä et al., in a study of 200 young adults [53]. Another study of 506 patients suggests a higher co-infection rate of 8% based on the detection of rising CRP levels at subsequent office visits; however, this is likely an overestimate since CRP measurements were made independent of microbiological confirmation [54] and it is well known that viral infections, such as influenza, parainfluenza, Epstein-Barr, herpes zoster, herpes simplex, and adenovirus may lead to substantial increases in CRP, often approaching 100 mg/mL [55,56,57,58]. Even though FebriDx cannot differentiate a rare co-infection, the 97% NPV suggests that the FebriDx test may supports a watchful waiting antibiotic strategy wherein if the patient were to clinically worsen over the subsequent 72 h after the initial testing, additional reflex microbiological testing or empiric antibiotic therapy could be considered.

Diagnostic tests should be interpreted in the context of the clinical picture and not independently as trauma, malnutrition, immunocompromised patients, or patients with underlying chronic disease may exhibit altered immune responses. In this study, FebriDx revealed a PPV of 63% for bacterial infection, which is largely related to the lower prevalence of bacterial infection compared to viral infection but is significantly higher than PPV’s reported in PCT studies evaluating ARI in primary care patients [59,60]. This suggests that approximately 30% of the FebriDx bacterial positive patients, which represents about 4% of the total ARI patients based on the prevalence, could receive an unnecessary antibiotic which is less than the current over prescription rate of 30% which is largely driven by over treating viral infections [2]. None of the FebriDx bacterial false positives occurred in patients with microbiologically confirmed viral infection. In this study, 38% of patients with microbiologically confirmed viral infection had an elevated CRP ≥ 20 mg/L and 17% had an elevated PCT ≥ 0.1 ng/ml which could contribute to overtreatment. Most URIs do not require antibiotic treatment but often receive unnecessary antibiotic treatment based on parent or patient pressure and physician fears of missing a serious bacterial infection. Davidson et al., showed that FebriDx altered clinical management decisions in 48% of patients with an ARI and reduced unnecessary antibiotic prescriptions by 80% [7]. The strength of the FebriDx test rests in its high NPV that reduces the likelihood of missing a clinically significant bacterial infection.

Defining a clinically significant bacterial infection that requires treatment is important to help drive antibiotic stewardship. Oropharyngeal and nasopharyngeal colonization occurs when microorganisms are detected without a significant associated systemic host response and may be affected by many host factors including immunosuppression, microbial competition, and or the use of antimicrobials. Molecular testing and microbial antigen detection may detect colonization or post-infectious shedding of respiratory pathogens with unknown clinical significance.

Arguably one of the most important bacterial pathogens in URI is GABHS. Treatment of GABHS may only be necessary when it is associated with a host response since it is very rare to have cases of rheumatic fever occur in patients without a systemic antibody response [47]. When GABHS is cultured from the oropharynx and is associated with an antibody response suggesting a true infection, CRP will elevate 80–90% of the time [46,61]. Patients with a URI and negative initial CRP test seldom show a rise in antibody titer [62]. Ninety-six percent of uninfected children have CRP < 10 mg/mL [63]. Putto et al., found that in examining 62 children with positive bacterial cultures, 89% showed a CRP elevated over 20 mg/L [37]. In a recent study of 621 adult patients with acute cough or fever, 20.5% of whom had radiographically confirmed pneumonia, Steurer et al., concluded that pneumonia could safely be excluded in patients with CRP values below 10 mg/L and in patients without dyspnea or daily fever with values between 11 and 50 mg/L [64]. Valkenburg et al., has shown that an anti-streptococcal antibody titer is more accurate than a throat culture in predicting therapeutic outcome [65]. Both CRP and PCT have been shown to elevate in URI [24,25]. In Europe, CRP > 20 mg/L is recommended by the National Institute for Health and Care Excellence (NICE) Pneumonia guidelines as a trigger for prescribing antibiotics [66]. Randomized controlled studies using CRP [67,68,69] and PCT [70] to guide antibiotic decisions have been shown to reduce antibiotic prescriptions without increased morbidity for both URI and lower respiratory tract infection (LRI). This inability to differentiate a local infection without an associated systemic host response from a clinically significant infection with an associated host response may result in unnecessary antibiotic prescriptions.

Patients with microbiological confirmation that lack a systemic immune response are more likely to have microbial colonization, DNA shedding from a previous infection, or a clinically insignificant local infection. Those patients without microbial confirmation and a limited immune response may represent a potentially less significant clinical case of MURI [36] Enrollment into the current study required that patients have a documented fever or reported fever ≥ 100.5 °F within the past 3 days, and new onset of cough or sore throat within the past 7 days. Since a confirmation of fever was only found in 13% (26/205), it is possible that some of these MURI patients were never febrile and were experiencing reactive airway disease or were presenting prior to, or significantly after, development of a clinically significant infection. The rate of MURI in the small confirmed febrile subset was 28% versus 62% for the complete trial and demonstrated 100% (4/4) bacterial sensitivity, 95% (21/22) specificity, 82% (9/11) viral sensitivity, and 87% (13/15) viral specificity. This suggests that clinical performance may be enhanced in the presence of a confirmed febrile episode.

Our study had limitations. First, as discussed above, there is no definitive reference standard for distinguishing between viral and bacterial respiratory infections; we used an algorithm-based approach to maximize reproducibility and validity of our reference standard, but some misclassification is possible. Second, we sought to enroll consecutive eligible patients, but due to practical constraints of shipping biological samples and availability of research staff, our population is best described as a convenience sample. Third, we did not include blood or sputum cultures in our testing for bacterial pathogens due to low anticipated yield for these tests in outpatients with URIs; we acknowledge that including a broader range of culture testing may have resulted in more bacterial identification. Fourth, we only captured information during a patient’s index visit; including follow-up information from later visits may have provided additional data useful for classifying URIs as viral or bacterial. Fifth, despite extensive testing, 62% of our URI patients were classified as having negative etiology by the reference standard; this is similar to prior studies demonstrating a large proportion of patients with acute respiratory infections do not have pathogens identifiable with currently-available tests [34,35]. Sixth, a larger dataset is needed to determine the role of FebriDx in the pediatric population.

## 5. Conclusions

FebriDx is a novel point-of-care test designed to identify systemic host immune responses to viral and bacterial infection through rapid measurement of MxA and CRP from a small sample of capillary blood. In this study, of diagnostic accuracy, the FebriDx test was sensitive and specific for identifying clinically-important viral and bacterial infections in febrile outpatients with URIs. FebriDx has potential to assist clinicians with rapidly distinguishing between viral and bacterial URIs and promoting antibiotic stewardship.

## Figures and Tables

**Table 1:** Participant characteristics.

Characteristic	URI Population (*n* = 205)	Asymptomatic Control Population (*n* = 163)
Female Gender, *n* (%)	123 (60.0)	90 (55.2)
Median age, years (IQR)	29 (15, 46)	44 (29, 55)
Age Groups, *n* (%)		
<18 years	56 (27.3)	5 (3.1)
18–50 years	112 (54.6)	99 (60.7)
>50 years	37 (18.1)	59 (36.2)
Race, *n* (%)		
White	142 (69.3)	134 (82.2)
Black	46 (22.4)	14 (8.6)
Other	17 (8.3)	15 (9.2)
Hispanic Ethnicity, *n* (%)	88 (42.9)	64 (39.3)
Median body temperature, °F (IQR)	101.5 (101.0, 102.1)	n/a
Median white blood cell count, thousand cells/mcL (IQR)	7.8 (5.9, 10.3)	n/a
Median serum procalcitonin, ng/mL (IQR)	<0.05 (<0.05, 0.07)	n/a
Median CRP, mg/L (IQR)	12.1 (3.8, 45.2)	2.0 (1.0, 4.0)
Median MxA, ng/mL (IQR)	4.9 (0.65, 20.2)	<1.0 (0.03, 2.0)

**Table 2:** Reference standard results.

Reference Standard Classification Criterion	*n*	Organisms Detected
**Bacterial**	**25**	
Throat culture positive for bacteria commonly causing pharyngitis and PCT ≥ 0.10 ng/mL	9	6 Group A *Streptococcus* 3 Group C *Streptococcus*
Throat culture positive for other bacteria and PCT ≥ 0.15 ng/mL	2	1 *Haemophilus parainfluenza* 1 *Enterobacter cloacae*
NP/OP PCR positive for atypical bacteria and PCT ≥ 0.10 ng/mL	1	1 *Chlamydophila pneumoniae*
Throat culture negative or contaminant, PCR negative, and PCT PCT ≥ 0.25 ng/mL	5	5 none
Throat culture negative or contaminant, PCR negative, and PCT between 0.15 ng/mL and 0.25 ng/mL, and WBC > 15 k or bands present	6	6 none
Physician panel over-read classified as bacterial after algorithm suggested negative	2	1 Group A *Streptococcus* 1 none
**Viral**	**53**	
NP/OP positive PCR for pathogenic virus	47	33 influenza 9 parainfluenza 3 RSV 1 hMPV 1 hMPV and parainfluenza
NP/OP positive PCR and IgM positive for EBV	3	3 EBV
Throat culture negative or contaminant, PCR negative or contaminant , and PCT between 0.15 ng/mL and 0.25 ng/mL, and WBC < 15 k, and no bands present	3	1 none 1 *Enterobacter gergoviae* 1 *Group B Streptococcus*
**Negative**	**127**	
Throat culture negative or contaminant, P/OP PCR negative, and PCT < 0.15 ng/mL	79	79 none
Throat culture positive for bacteria commonly causing pharyngitis and PCT < 0.10 ng/mL	11	10 Group A *Streptococcus* 1 Group C *Streptococcus*
Throat culture positive for other bacteria and PCT < 0.15 ng/mL	37	18 *S. aureus* 3 Group B *Streptococcus* 3 *Klebsiella pneumoniae* 3 Group B *Strep* and *S. aureus* 3 *Enterobacter* sp. 2 *Streptococcus pneumoniae* 2 *Proteus mirabilis* 1 *Citrobacter freundi* 1 *M. catarrhalis* and *S. aureus* 1 *Anicetobacter* sp and *S. aureus*

**Table 3:** Cross tabulation of FebriDx and reference standard results for the upper respiratory tract infection population.

FebriDx Test		Reference Standard			Total
Test	FebriDx Result	Bacterial	Viral	Negative	
**FebriDx**	Bacterial	20	1	11	32
	Viral	1	*46*	25	72
	Negative	4	6	*91*	101
	Total	25	53	127	205

**Table 4:** Diagnostic accuracy of FebriDx for identifying (A) bacterial and (B) viral infections compared to the reference standard in the upper respiratory tract infection population.

Population		Total *n*	Bacterial/Viral by Reference Standard, *n* (%)	FebriDx Test Characteristics			
				Sens (%) (95% CI)	Spec (%) (95% CI)	PPV (%) (95% CI)	NPV (%) (95% CI)
**(A) Bacterial**							
Full URI population		205	25 (12%)	80% (59–93%)	93% (90–97%)	63% (45–79%)	97% (94–99%)
	Age < 18	56	5 (8.9%)	60% (16–95%)	100% (94–100%) *	100% (30–100%) *	96% (88–100%)
	Age 18–50	112	13 (12%)	85% (56–98%)	90% (84–96%)	55% (33–77%)	98% (93–100%)
	Age > 50	37	7 (19%)	86% (43–100%)	90% (75–98%)	67% (31–93%)	96% (83–100%)
**(B) Viral**							
Full URI population		205	53 (26%)	87% (75–95%)	83% (77–89%)	64% (53–75%)	95% (90–98%)
	Age <18	56	25 (45%)	96% (80–100%)	55% (37–73%)	63% (47–78%)	94% (73–100%)
	Age 18–50	112	21 (19%)	76% (53–92%)	87% (80–93%)	57% (38–76%)	94% (87–98%)
	Age >50	37	7 (19%)	86% (42–100%)	100% (89–100%)*	100% (54–100%) *	97% (83–100%)

* A 97.5% one-sided lower bound of the confidence interval is provided for point estimates of 100%.

## Supplementary Materials

The following are available online at http://www.mdpi.com/2077-0383/6/10/94/s1. Supplemental Table 1. Eligibility criteria for the upper respiratory tract study population; Supplemental Table 2. Eligibility criteria for the asymptomatic control study population.

## References

[1] Shapiro D.J., Hick L.A., Pavia A.T., Hersh A.L. (2014). Antibiotic prescribing for adults in ambulatory care in the USA, 2007–2009. J. Antimicrob. Chemother..

[2] Fleming-Dutra K.E., Hersh A.L., Shapiro D.J. (2016). Prevalence of inappropriate antibiotic prescriptions among US ambulatory care visits, 2010–2011. JAMA.

[3] Dellit T.H., Owens R.C., McGowan J.E., Gerding D.N., Weinstein R.A., Burke J.P., Huskins W.C., Paterson D.L., Fishman N.O., Carpenter C.F. (2007). Infectious Diseases Society of America and the Society for Healthcare Epidemiology of America guidelines for developing an institutional program to enhance antimicrobial stewardship. Clin. Infect. Dis..

[4] Centers for Disease Control and Prevention Get Smart: Know when Antibiotics Work. http://www.cdc.gov/getsmart/community/index.html.

[5] Caliendo A.M., Gilbert D.N., Ginocchio C.C., Hanson K.E., May L., Quinn T.C., Tenover F.C., Alland D., Blaschke A.J., Bonomo R.A. (2013). Better tests, better care: Improved diagnostics for infectious diseases. Clin. Infect. Dis..

[6] FebriDx RPS Diagnostics. https://www.rpsdetectors.com/in/products/febridx/.

[7] Davidson M. (2017). FebriDx Point-of-Care Testing to Guide Antibiotic Therapy for Acute Respiratory Tract Infection in UK Primary Care: A Retrospective Outcome Analysis. J. Infect. Dis. Prev. Med..

[8] Engelmann I., Dubos F., Lobert P.E., Houssin C., Degas V., Sardet A., Decoster A., Dewilde A., Martinot A., Hober D. (2015). Diagnosis of viral infections using myxovirus resistance protein A (MxA). Pediatrics.

[9] Simon L., Gauvin F., Amre D.K., Saint-Louis P., Lacroix J. (2004). Serum procalcitonin and c-reactive protein levels as markers of bacterial infection: A systematic review and meta-analysis. Clin. Infect. Dis..

[10] Kawamura M., Kusano A., Furuya A., Hanai N., Tanigaki H., Tomita A., Horiguchi A., Nagata K., Itazawa T., Adachi Y. (2012). New sandwich-type enzyme-linked immunosorbent assay for human MxA protein in a whole blood using monoclonal antibodies against GTP-binding domain for recognition of viral infection. J. Clin. Lab. Anal..

[11] Schuetz P., Briel M., Mueller B. (2013). Clinical outcomes associated with procalcitonin algorithms to guide antibiotic therapy in respiratory tract infections. JAMA.

[12] Musher D.M., Bebko S.P., Roig I.L. (2014). Serum procalcitonin level, viral polymerase chain reaction analysis, and lower respiratory tract infection. J. Infect. Dis..

[13] Self W.H., Williams D.J., Zhu Y.W., Ampofo K., Pavia A.T., Chappell J.D., Hymas W.C., Stockmann C., Bramley A.M., Schneider E. (2016). Respiratory viral detection in children and adults: Comparing asymptomatic controls and patients with community-acquired pneumonia. J. Infect. Dis..

[14] Jansen R.R., Wieringa J., Koekkoek S.M., Visser C.E., Pajkrt D., Molenkamp R., de Jong M.D., Schinkel J. (2011). Frequent detection of respiratory viruses without symptoms: Toward defining clinically relevant cutoff values. J. Clin. Microbiol..

[15] Jartti T., Jartti L., Peltola V., Maris M., Ruuskanen O. (2008). Identification of respiratory viruses in asymptomatic subjects: Asymptomatic respiratory viral infections. Pediatr. Infect. Dis. J..

[16] FilmArray Respiratory Panel BioMerieux. http://www.biomerieux-diagnostics.com/filmarrayr-respiratory-panel.

[17] EBV-VCA IgM Enzyme Immunoassay Test Kit Diamedix. http://diamedix.com/wp-content/uploads/2015/10/PI-EBVVCAIgM-720610-Rev7-June-15.pdf.

[18] BRAHMS PCT Sensitive KRYPTOR Thermo Fisher Scientific. http://www.procalcitonin.com/.

[19] Korppi M., Kroger L., Laitinen M. (1993). White blood cell and differential counts in acute respiratory viral and bacterial infections in children. Scand. J. Infect. Dis..

[20] Casey J.R., Marsocci S.M., Murphy M.L., Francis A.B., Pichichero M.E. (2003). White blood cell count can aid judicious antibiotic prescribing in acute upper respiratory infections in children. Clin. Pediatr..

[21] Burkhardt O., Ewig S., Haagen U., Giersdorf S., Hartmann O., Wegscheider K., Hummers-Pradier E., Welte T. (2010). Procalcitonin guidance and reduction of antibiotic use in acute respiratory tract infection. Eur. Respir. J..

[22] Briel M., Schuetz P., Mueller B., Young J., Schildm U., Nusbaumer C., Périat P., Bucher H.C., Christ-Crain M. (2008). Procalcitonin-guided antibiotic use vs a standard approach for acute respiratory tract infections in primary care. Arch. Intern. Med..

[23] Dandona P., Nix D., Wilson M.F., Aljada A., Love J., Assicot M. (1994). Procalcitonin increase after endotoxin injection in normal subjects. J. Clin. Endocrinol. Metab..

[24] Stolz D., Christ-Crain M., Gencay M.M., Bingisser R., Huber P.R., Müller B., Tamm M. (2006). Diagnostic value of signs, symptoms and laboratory values in lower respiratory tract infection. Swiss Med. Wkly..

[25] Elsammak M., Hanna H., Ghazal A., Edeen F.B., Kandil M. (2006). Diagnostic value of serum procalcitonin and C-reactive protein in Egyptian children with streptococcal tonsillopharyngitis. Pediatr. Infect. Dis. J..

[26] Christensen A.M., Thomsen M.K., Ovesen T., Klug T.E. (2014). Are procalcitonin or other infection markers useful in the detection of group A streptococcal acute tonsillitis?. Scand. J. Infect. Dis..

[27] Landis J.R., Koch G.G. (1977). The measurement of observer agreement for categorical data. Biometrics.

[28] Reichenheim M.E. (2004). Confidence intervals for the kappa statistic. Stata J..

[29] Stewart E.H., Davis B., Clemans-Taylor B.L., Littenberg B., Estrada C.A., Centor R.M. (2014). Rapid antigen Group A Streptococcus Test to diagnose pharyngitis: A systematic review and meta-analysis. PLoS ONE.

[30] Dingle T.C., Abbott A.N., Fang F.C. (2014). Reflexive culture in adolescents and adults with Group A Streptococcal pharyngitis. Clin. Infect. Dis..

[31] Inverness Medical: BinaxNow Influenza A&B CLIA Waived (Package Insert Training Packet). http://www.amms.net/images/healthcare_industry_clia_binax_now_influenza_test_2009.pdf.

[32] Uyeki T.M., Prasad R., Vukotich C., Stebbins S., Rinaldo C., Ferng Y.H., Morse S.S., Larson E.L., Aiello A.E., Davis B. (2009). Low sensitivity of rapid diagnostics tests for influenza. Clin. Infect. Dis..

[33] Self W.H., McNaughton C.D., Grijalva C.G., Zhu Y., Chappell J.D., Williams J.V., Talbot H.K., Shay D.K., Griffin M.R. (2012). Diagnostic performance of the BinaxNow Influenza A&B rapid antigen test in ED patients. Am. J. Emerg. Med..

[34] Andreola B., Bressan S., Callegaro S., Liverani A., Plebani M., Da Dalt L. (2007). Procalcitonin and C-reactive protein as diagnostic markers of severe bacterial infections in febrile infants and children in the emergency department. Pediatr. Infect. Dis. J..

[35] Bafadhel M., Clark T.W., Reid C., Medina M.J., Batham S., Barer M.R., Nicholson K.G., Brightling C.E. (2011). Procalcitonin and c-reactive protein in hospitalized adult patients with community-acquired pneumonia or exacerbation of asthma or COPD. Chest.

[36] Sambursky R., Shapiro N.I. (2015). Evaluation of a combined MxA and CRP point-of-care immunoassay to identify viral and/or bacterial immune response in patients with acute febrile respiratory infection. Eur. Clin. Respir. J..

[37] Putto A., Ruuskanen O., Meurman O., Ekblad H., Korvenranta H., Mertsola J., Peltola H., Sarkkinen H., Viljanen M.K., Halonen P. (1986). C reactive protein in the evaluation of febrile illness. Arch. Dis. Child..

[38] Hatherill M., Tibby S.M., Sykes K., Turner C., Murdoch I.A. (1999). Diagnostic markers of infection: Comparison of procalcitonin with c reactive protein and leukocyte count. Arch. Dis. Child..

[39] Roers A., Hochkeppel H.K., Horisberger M.A., Hovanessian A., Haller O. (1994). MxA gene expression after live virus vaccination: A sensitive marker for endogenous type I interferon. J. Infect. Dis..

[40] Staeheli P., Pitossi F., Pa vlovic J. (1993). Mx proteins: GTPases with antiviral activity. Trends Cell Biol..

[41] Staeheli P. (1990). Interferon induced proteins and the antiviral state. Adv. Virus Res..

[42] Chieux V., Hober D., Harvey J., Lion G., Lucidarme D., Forzy G., Duhamel M., Cousin J., Ducoulombier H., Wattré P. (1998). The MxA protein levels in while blood lysates of patients with various viral infections. J. Virol. Methods.

[43] Jain S., Self W.H., Wunderink R.G., Fakhran S., Balket R., Bramley A.M., Reed C., Grijalva C.G., Anderson E.J., Courtney M. (2015). Community-acquired pneumonia requiring hospitalization among US adults. N. Engl. J. Med..

[44] Branche A.R., Walsh E.E., Vargas R., Hulbert B., Formica M.A., Baran A., Peterson D.R., Falsey A.R. (2015). Serum procalcitonin measurement and viral testing to guide antibiotic use for respiratory infections in hospitalized adults: A randomized controlled trial. J. Infect. Dis..

[45] Bosch A.A., Biesbroek G., Trzcinski K., Sanders E.A., Bogaert D. (2013). Viral and bacterial interactions in the upper respiratory tract. PLoS Pathog..

[46] Kaplan E.L., Top F.H., Dudding B.A., Wannamaker L.W. (1971). Diagnosis of streptococcal pharyngitis: Differentiation of active infection from the carrier state in the symptomatic child. J. Infect. Dis..

[47] Kaplan E.L. (1980). The group A streptococcal upper respiratory tract carrier state: An enigma. J. Pediatr..

[48] Gerber M.A., Randolph M.F., Mayo D.R. (1988). The group A streptococcal carrier state. A reexamination. Am. J. Dis. Child..

[49] Komaroff A.L., Pass T.M., Aronson M.D., Ervin C.T., Cretin S., Winickoff R.N., Branch W.T. (1986). The prediction of streptococcal pharyngitis in adults. J. Gen. Intern. Med..

[50] Gerber M.A., Randolph M.F., Chanatry J., Wright L.L., DeMeo K.K., Anderson L.R. (1986). Antigen detection test for streptococcal pharyngitis: Evaluation of sensitivity with respect totrue infection. J. Pediatr..

[51] Nussinovitch M., Finkelstein Y., Amir J., Varsano I. (1999). Group A beta-hemolytic streptococcal pharyngitis in preschool children aged 3 months to 5 years. Clin. Pediatr..

[52] Ivaska L., Niemelä J., Lempainen J., Österback R., Warism M., Vuorinen T., Hytönen J., Rantakokko-Jalava K., Peltola V. (2017). Aetiology of febrile pharyngitis in children: Potential of myxovirus resistance protein A (MxA) as a biomarker of viral infection. J. Infect..

[53] Mäkelä M.J., Puhakka T., Ruuskanen O., Leinonen M., Saikku P., Kimpimäki M., Blomqvist S., Hyypiä T., Arstila P. (1998). Viruses and bacteria in the etiology of the common cold. J. Clin. Microbiol..

[54] Lingard H., Zehetmayer S., Maier M. (2008). Bacterial superinfection in upper respiratory tract infections estimated by increases in CRP values: A diagnostic follow-up in primary care. Scand. J. Prim. Health Care.

[55] Salonen E.M., Vaheri A. (1981). C-Reactive protein in acute viral infections. J. Med. Virol..

[56] Sarov I., Shainkin-Kestenbaum R., Zimlichman R., Winikoff Y., Chaimovitz C., Pras M. (1982). Serum amyloid A levels in patients with infections due to cytomegalovirus, varicella-zoster virus and Herpes simplex virus. J. Infect. Dis..

[57] Whicher J.T., Chambers R.E., Higginson J., Nashef L., Higgins P.G. (1985). Acute phase response of serum amyloid A protein and C reactive protein to the common cold and influenza. J. Clin. Pathol..

[58] Melbye H., Hvidsten D., Holm A., Nordbø S.A., Brox J. (2004). The course of C-reactive protein response in untreated upper respiratory tract infection. Br. J. Gen. Pract..

[59] Holm A., Pedersen S.S., Nexoe J., Obel N., Nielsen L.P., Koldkjaer O., Pedersen C. (2007). Procalcitonin versus C-reactive protein for predicting pneumonia in adults with lower respiratory tract infection in primary care. Br. J. Gen. Pract..

[60] Kutz A., Briel M., Christ-Crain M., Stolz D., Bouadma L., Wolff M., Kristoffersen K.B., Wei L., Burkhardt O., Welte T. (2015). Prognostic value of procalcitonin in respiratory tract infections across clinical settings. Crit. Care.

[61] Kaplan E.L., Wannamaker L.W. (1977). C-reactive protein in streptococcal pharyngitis. Pediatrics.

[62] Wannamaker L.W., Ayoub E.M. (1960). Antibody titers in acute rheumatic fever. Circulation.

[63] Hanson L.A., Jodal U., Sabel K.G., Wadsworth C. (1983). The diagnostic value of C-reactive protein. Pediatr. Infect. Dis..

[64] Steurer J., Held U., Spaar A., Bausch B., Zoller M., Hunziker R., Bachmann L.M. (2011). A decision aid to rule out pneumonia and reduce unnecessary prescriptions of antibiotics in primary care patients with cough and fever. BMC Med..

[65] Valkenburg H.A., Haverkorn M.J., Goslings W.R. (1971). Streptococcal pharyngitis in the general population. II. The attack rate of rheumatic fever and acute glomerulonephritis in patients. J. Infect. Dis..

[66] National Clinical Guideline Centre (UK) (2014). Pneumonia: Diagnosis and management of community and hospital acquired pneumonia in Adults. Clin. Guidel..

[67] Huang Y., Chen R., Wu T., Wei X., Guo A. (2013). Association between point-of-care CRP testing and antibiotic prescribing in respiratory tract infections: A systematic review and meta-analysis of primary care studies. Br. J. Gen. Pract..

[68] Cooke J., Butler C., Hopstaken R., Dryden M.S., McNulty C., Hurding S., Moore M., Livermore D.M. (2015). Narrative review of primary care point-of-care testing (POCT) and antibacterial use in respiratory tract infection (RTI). BMJ Open Respir. Res..

[69] Cals J.W.L., Butler C.C., Hopstaken R.M., Hood K., Dinant G.-J. (2009). Effect of point of care testing for C reactive protein and training in communication skills on antibiotic use in lower respiratory tract infections: Cluster randomised trial. BMJ.

[70] Schuetz P., Chiappa V., Briel M., Greenwald J.L. (2011). Procalcitonin algorithms for antibiotic therapy decisions: A systematic review of randomized controlled trials and recommendations for clinical algorithms. Arch. Intern. Med..

