# Supplementary material for: Diagnostic Accuracy of FebriDx: A Rapid Test to Detect Immune Responses to Viral and Bacterial Upper Respiratory Infections
Source: J Clin Med. 2017 Oct 7;6(10):94. doi: 10.3390/jcm6100094 (PMC5664009; doi:10.3390/jcm6100094)
Supplement: Supplementary file 1 [file jcm-06-00094-s001.docx]

**SUPPLEMENTAL MATERIALS**

**Supplemental Table 1.** Eligibility criteria for the upper respiratory tract study population.

| **Upper Respiratory Tract Infection Population Eligibility Criteria** | | |
| --- | --- | --- |
| **Inclusion Criteria** | | |
|  | 1. Age > 1 year | |
|  | 1. Exhibited or reported temperature (oral or tympanic) ≥ 100.5ºF within 3 days of presentation | |
|  | 1. New onset within the previous 7 days of either: | |
|  |  | 1. significant cough, or |
|  |  | 1. sore throat plus ≥ 1 of the following signs: swollen or tender anterior cervical nodes; tonsillar erythema; tonsillar swelling; tonsillar exudate; peritonsillar petechial hemorrhages |
| **Exclusion Criteria** | | |
|  | 1. Interferon therapy in the last 30 days | |
|  | 1. Immunocompromised state or taking immunosuppressive or chemotherapeutic medications in the last 30 days | |
|  | 1. Antibiotics or antiviral therapy in the last 30 days | |
|  | 1. Live viral immunization in the last 30 days | |
|  | 1. Significant trauma or burns (> 5% total body surface are or full thickness) in the last 30 days | |
|  | 1. Major surgery (requiring intravenous anesthesia or respiratory assistance) in the last 30 days | |
|  | 1. Chronic fever greater than 7 days | |

**Supplemental Table 2.** Eligibility criteria for the asymptomatic control study population.

| **Asymptomatic Control Population Eligibility Criteria** | |
| --- | --- |
| **Inclusion Criteria** | |
|  | 1. Age > 1 year |
|  | 1. Presentation to a primary care clinic, urgent care clinic, or emergency department for a noninfectious illness |
| **Exclusion Criteria** | |
|  | 1. Fever ≥ 100.5ºF (oral or tympanic) in the last 14 days |
|  | 1. Cough |
|  | 1. Chills |
|  | 1. Dyspnea |
|  | 1. Fatigue |
|  | 1. Purulent sputum |
|  | 1. Pleuritic chest pain |
|  | 1. Nasal congestion |
|  | 1. Rhinorrhea |
|  | 1. Sore throat |
|  | 1. Autoimmune or rheumatologic disease |
|  | 1. Interferon therapy in the last 30 days |
|  | 1. Immunocompromised state or taking immunosuppressive or chemotherapeutic medications in the last 30 days |
|  | 1. Antibiotics or antiviral therapy in the last 30 days |
|  | 1. Live viral immunization in the last 30 days |
|  | 1. Significant trauma or burns (> 5% total body surface are or full thickness) in the last 30 days |
|  | 1. Major surgery (requiring intravenous anesthesia or respiratory assistance) in the last 30 days |
|  | 1. Myocardial infarction or stroke in the last 30 days |
|  | 1. Chronic bacterial infection or osteomyelitis |
|  | 1. Known chronic viral infection such as HIV, HCV, HBV, or CMV |
|  | 1. Active tuberculosis |
|  | 1. Acute or chronic (> 30 days) diarrhea and/or vomiting |
|  | 1. Urinary tract symptoms in the last 14 days |
